# The Dynamic Conformational Cycle of the Group I Chaperonin C-Termini Revealed via Molecular Dynamics Simulation

**DOI:** 10.1371/journal.pone.0117724

**Published:** 2015-03-30

**Authors:** Kevin M. Dalton, Judith Frydman, Vijay S. Pande

**Affiliations:** 1 Biophysics Program, Stanford University, Stanford, California, United States of America; 2 Department of Biology, Stanford University, Stanford, California, United States of America; 3 Department of Chemistry, Stanford University, Stanford, California, United States of America; University of South Florida College of Medicine, UNITED STATES

## Abstract

Chaperonins are large ring shaped oligomers that facilitate protein folding by encapsulation within a central cavity. All chaperonins possess flexible C-termini which protrude from the equatorial domain of each subunit into the central cavity. Biochemical evidence suggests that the termini play an important role in the allosteric regulation of the ATPase cycle, in substrate folding and in complex assembly and stability. Despite the tremendous wealth of structural data available for numerous orthologous chaperonins, little structural information is available regarding the residues within the C-terminus. Herein, molecular dynamics simulations are presented which localize the termini throughout the nucleotide cycle of the group I chaperonin, GroE, from Escherichia coli. The simulation results predict that the termini undergo a heretofore unappreciated conformational cycle which is coupled to the nucleotide state of the enzyme. As such, these results have profound implications for the mechanism by which GroE utilizes nucleotide and folds client proteins.

## Introduction

Many cellular proteins cannot fold on their own and often become trapped in non-native intermediate conformations. Nature has evolved a variety of molecular chaperones that bind to and induce folding of these intermediates in an ATP dependent manner[1]. The chaperonins are one such class of molecular chaperone and are essential in nearly all characterized organisms. Chaperonins fold many different substrates and have been estimated to routinely interact with as much as 10% of the proteome[2–4].

The chaperonins are subdivided into two phyla, termed Group I and Group II chaperonins[5]. Group I chaperonins are found in prokaryotes as well as the eukaryotic organelles derived from endosymbiotic events, chloroplasts and mitochondria. The archetypal Group I chaperonin is the GroE system from *Escherichia coli*.

**Fig 1:**
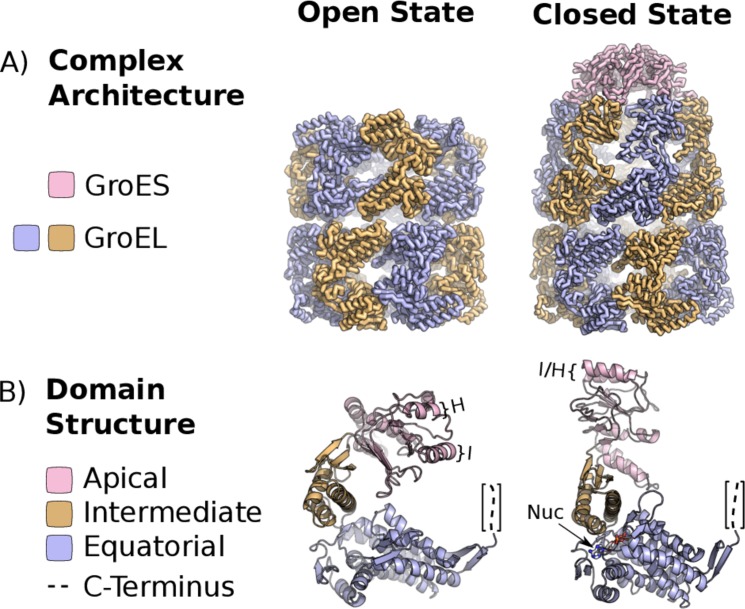
The Molecular Architecture of GroE. The crystal structure of the GroE chaperonin from Escherichia coli in the open, ATP-bound (PDBID: 1KP8) and closed (PDBID: 1AON) states. The full complex and single subunits are depicted in panels A and B respectively. Helices I and H as well as the nucleotide binding pocket are indicated in B. Structures were rendered in PyMOL.

GroE consists of the GroEL complex, an (α_7_)_2_ homoligomer with a dual-ring topology[6], and its homoheptameric cofactor, GroES, which acts as a lid for the GroEL folding chamber[7] (Fig. 1A). GroEL monomers are 548 residues in length and have 3 domains (Fig. 1B). The equatorial domain is located at the ring interface and contains the ATP binding site[7,8]. The intermediate domain acts as a hinge upon which the apical domain pivots and contains the residues which form the apical surface of the nucleotide binding pocket (Fig. 1B). The apical domain binds to substrate proteins and to the GroES complex[8]. The apical domains of a given GroEL ring are competent to bind unfolded client proteins in the apo or ATP-bound configurations. Subsequent to substrate binding, GroES binding displaces the client protein in an ATP dependent manner, injecting it into the center of the chaperonin ring where it may fold in isolation. Substrate displacement is followed by nucleotide hydrolysis which is essential for GroES dissociation. GroES dissociation and substrate release complete the cycle.

Substrate encapsulation is the hallmark of the chaperonin nucleotide cycle which differentiates it from other molecular chaperones[1]. Encapsulation is essential for efficient folding of substrates. There are many proposed mechanisms by which encapsulation improves folding yields[9]. The most obvious mechanism is the prevention of intermolecular aggregation by isolating the substrate from the cellular protein pool which may be the chaperonin's lone mechanism for a portion of its substrates[10]. Nonetheless, it has been demonstrated that the microenvironment of the GroE cavity accelerates the folding of other substrates yielding faster folding rates than are observed for the same protein in free solution[11]. This rate enhancement appears to originate from a variety of sources. Chief among these is the steric exclusion of extended conformers in the unfolded ensemble[12,13], modulation of the solvent environment by the cavity walls[14–16] and an unfolding event early in the encapsulation process[17]. The mechanism by which the three major structural domains of GroEL function together to give rise to this closed, encapsulated state is well understood.

In addition to the three well characterized domains, GroEL possesses a C-terminal region which is a disordered segment of 23 amino acids following Pro-525. A short hydrophilic motif, P525—KNDAAD—L532, followed by an approximate repeat of the Gly-Gly-Met tripeptide comprise this terminal segment. Numerous structures of chaperonins containing terminal repeats have been solved representing both the open and closed configurations[6,7,18–27]. Despite the high resolution of these structures, the C-termini do not appear in the electron density maps indicating that they exist in an ensemble of conformations. In archaeal Group II chaperonins, the role of the disordered C-termini is clear in that they determine the thermostability of the complex[28]. By contrast, deletion of the disordered region at the C-terminus of GroEL does not seem to perturb the stability of the complex or demonstrate a growth defect in vivo[29]. Consequently, the role of the GroEL C-termini in the chaperonin mechanism is rather subtle. Deletion of the C-terminal repeats in GroEL modestly decreases both the ATPase rate[30,31] and the folding activity[32] of the enzyme. Furthermore, disulfide crosslinking of substrates to GroEL demonstrated that the repeats are in close proximity to bound substrate prior to encapsulation[33]. The interaction of bound substrates with the C-terminus demonstrated by in vitro crosslinking[33] has been corroborated by a proteomic study which demonstrated differential substrate specificity for two variants of GroEL with different C-terminal sequences[34]. Taken together these results indicate that the GGM repeats play a role in substrate recognition. More recent work supports the hypothesis that the C-termini of GroEL are involved in substrate encapsulation in addition to binding Specifically, this work demonstrated that the C-termini interact with the substrate protein RuBisCO during the encapsulation process, enhance the yield of substrate encapsulation[35], and remodel the substrate's conformation before and throughout encapsulation[17].

Despite this sizable body of biochemical work, it is still unclear how the termini fit into the overall structural mechanism of GroE. Why does their deletion perturb the ATPase and folding rates of the enzyme?

## Results

In order to elucidate the mechanism by which the C-terminus influences the activity of GroEL, we sought structural information about the GGM repeats via molecular dynamics simulations. In this study, the open, ATP-bound (PDB ID: 1KP8)[36] and closed ADP-bound (PDB ID: 1AON)[7] structures of GroE were simulated in implicit solvent for a total of 190 ns each. In order to limit the computational complexity of the simulations, the N-terminal 523 amino acids of GroEL as well as all the residues of GroES were frozen throughout the simulations. The two simulations contained three distinct types of chaperonin rings corresponding to three intermediates of the GroE nucleotide cycle. Both rings of the open state simulation, based on PDBID: 1KP8, correspond to an ATP bound ring which is competent to bind unfolded substrates. The closed state simulation based on PDBID: 1AON contained two types of chaperonin rings. In particular, the trans-ring corresponds to an apo ring lacking any bound nucleotide which is also competent to bind substrate. By contrast, the cis-ring of the same simulation is bound by GroES as well as ADP. The cis-ring has thereby formed a folding chamber in which substrates may be encapsulated. The three ring types will be referred to as the holo for ATP-bound, apo for the trans-ring of the closed state simulation, and closed for the GroES-bound ring. It is pertinent to note that the holo and apo rings have very similar conformations exhibiting a 1.4 Å RMSD (S4 Fig.).

**Fig 2:**
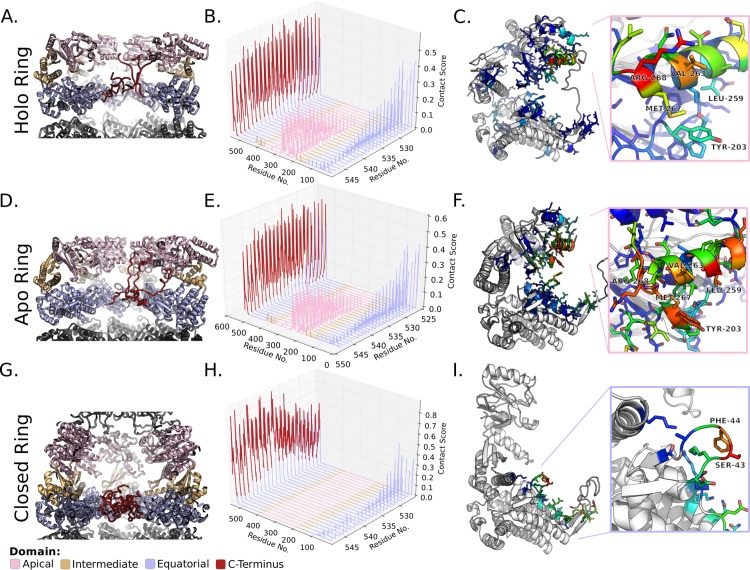
Characterization of C-terminal Contacts in GroE Simulations. Residue contacts of the GroEL C-terminus from 190 ns of implicit solvent molecular dynamics simulations. A,D,G) Representative snapshots of the the GroE simulations colored by domain as indicated. B,E,H) Residue contacts between the terminal residues and the rest of the chaperonin residues from the open and closed state simulations. Higher values represent more frequent contacts. C,F,I) Heat maps of the terminal residue's (M548) contacts mapped onto representative monomer from the simulations. The monomer structures derive from the holo enzyme simulation (C), the trans-ring of the GroES bound (F), and the cis-ring of the GroES bound (I) simulations. The insets represent helix I (C,F) or the stem loop (I). C) A heat map of the holo ring's M548 contacts mapped onto a representative snapshot from the simulation. Inset: view of the apical domain helix I colored by M548 contact frequency. In panels C, F, & I contacts between M548 and other tail residues have been omitted by setting their values as zero. Structures were rendered in PyMOL.

Analysis of the GroE Langevin dynamics simulations yielded very similar C-terminal conformations between the apo and holo rings. However, the conformation of the termini differed markedly for the closed ring. In order to represent the difference in conformation between the states of GroE, contact maps were constructed with g_mdmat from Gromacs[37] (S1, S2 Figs.). The C-terminal 23 amino acids which form the GGM repeats were then investigated in the context of these contact maps (Fig. 2). The conformation of these residues differs strikingly between closed ring and two rings not bound by GroES. In both simulations, the repeats showed the strongest contact with other residues within the termini (Fig. 2 B,E,H red) which is unsurprising given the proximity between these residues in the primary sequence. Nonetheless, the apo and holo rings' termini showed considerable interaction with residues within the apical domain of the chaperonin (Fig. 2 B,E pink). The most significant interactions are between the terminus and hydrophobic residues lining the inner face of the apical domain's helix I (Fig. 1C) which has been implicated in substrate binding by biochemical and structural studies. Particularly strong interactions are observed for residues R268, M267, V263 & L259 (Fig. 2 C,F). Notably, these hydrophobic residues are thought to directly contact the substrate, and mutations at V263 and L259 have been shown to perturb the binding of both substrate and GroES to GroEL[8]. The validity of the apo and holo ring ensembles is bolstered by a recent cryoelectron microscopy study[35] which demonstrated complex electron density in the trans-ring of the singly-closed GroEL/ES. The authors of this study speculated that the trans-ring density may originate from extended C-termini. By contrast, in the closed state, helix I of the apical domain is retracted upward and is occupied by GroES. Hence, the C-termini no longer contact the apical domain of the active folding chamber and only interact weakly with residues outside of the flexible tail. These interactions are predominantly restricted to the stem loop of the equatorial domain (Fig. 2 I), which is proximal to the nucleotide binding site.

## Discussion

**Fig 3:**
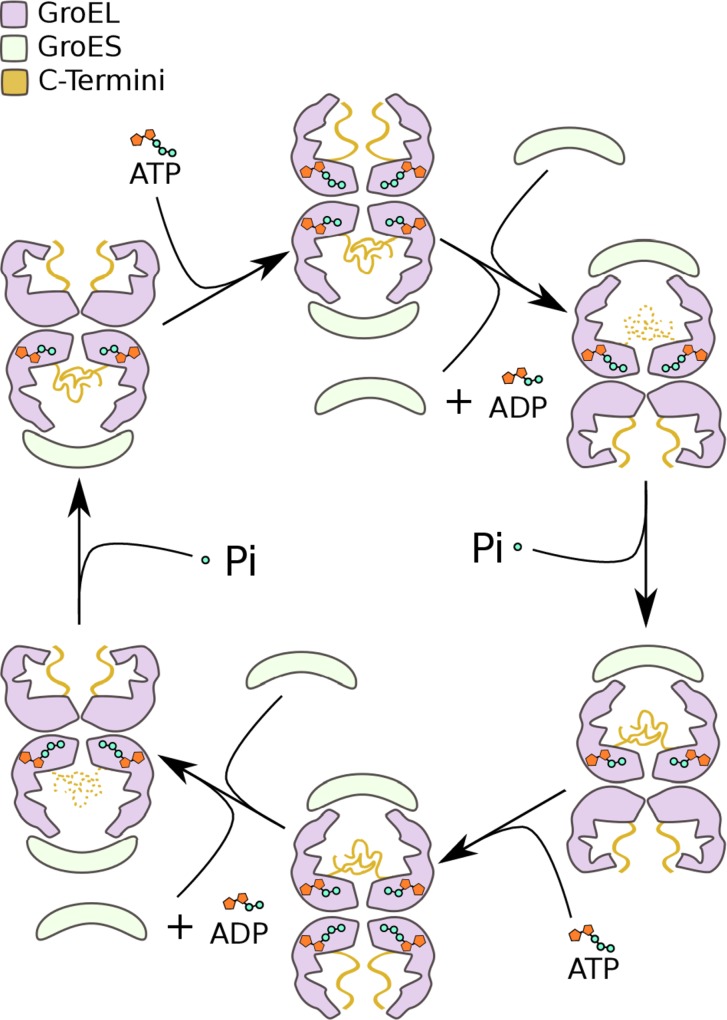
The Dynamic Conformational Cycle of the GroEL C-Termini. Schematic representation of the GroE nucleotide cycle emphasizing the C-terminal conformational dynamics/cycle. The conformational cycle of the C-terminal tails is indicated throughout the group I chaperonin nucleotide cycle. States with the C-termini rendered as dashes are hypothetical while those rendered with solid lines represent rings contained in our simulations.

The simulations presented herein predict a new conformational cycle within the group I chaperonin which has proven inaccessible to classical structural biology. The cycle, schematized in Fig. 3, summarizes the manner in which the GroE ATPase cycle impacts the structure of the C-termini and how the termini alter the chemical environment within the cavity throughout. This work provides a plausible conformational ensemble for the C-termini of ATP-bound rings, apo trans-rings and of ADP-bound cis-rings. No attempt has been made to extensively model the intermediate conformations in this cycle owing to a lack of atomic level structural data on these states. C-terminal conformations indicated by dashed lines in Fig. 3 are at present hypothetical and not contained within the simulation data. In the context of this model, it is possible to reconcile a portion of the biochemical data regarding the chaperonin C-termini.

Irrespective of the particular structural mechanism, the simulations predict that the conformation of the C-termini must transition along the pathway between the apo and closed, cis-ring conformations. While the precise details of when or how these transitions occur are yet to be determined, the termini must necessarilly be involved in the transition states of the opening and closing conformational changes. It should thus be expected that their deletion would effect the energy of the transition states. Thereby, the deletion of the termini should influence the closing and opening rates of GroE. The involvement of the termini in the transition between the open and closed conformations clarifies why deletion of the termini influences the ATPase rate of the enzyme[30,31].

In addition to the termini's involvement in the nucleotide cycle, the simulations predict that the C-termini play a role in substrate binding. It has already been shown that the C-termini of GroEL engage the substrate upon binding and that they are structurally and biochemically involved in the encapsulation process[35,17]. However, the concentration of the C-termini at helix I (Fig. 2 C,F) in the apo and holo rings, predicts that the GGM repeats may occupy the substrate binding site prior to substrate binding. The apical localization of the termini therefore has implications for the manner in which GroE recognizes substrates. As they occupy the binding site, the GGM repeats must modulate the affinity of the GroEL apical domains for clients. Biochemical evidence[35] indicates that the C-terminus of GroEL contributes to the effective encapsulation of substrates. This suggests that the GGM repeats increase the affinity of the chaperonin for unfolded clients in the apo state as well as the intermediate states leading to closure of the folding chamber. Based on the apo state conformational ensemble, the termini likely increase the affinity by providing a secondary substrate binding site. It is tempting to speculate that this secondary binding site may possess broader specificity than the apical domain Helix I owing to its low sequence complexity and lack of secondary structure. Consequently the GGM repeats may ensure that the chaperonin interacts with a broader range of misfolded clients than may otherwise be possible. The precedent for modulation of the chaperonin substrate pool by the termini has been demonstrated proteomically[34] indicating that substrate binding is one important function of the GGM repeats.

The isolation of the termini at the bottom of the folding chamber in the closed state does not portend an active role for the termini in the foldase activity[11] of the GroE cavity. However, these simulations were carried out in the absence of substrate, and it is impossible to know precisely how an encapsulated substrate may alter the conformational ensemble of the C-termini. Nevertheless, it has been demonstrated that the deletion of the GroEL C-termini alters the folding trajectory of RuBisCO[17]. Therefore it is clear that the C-termini can influence substrate conformation potentially by steric interaction or alternatively by altering the electrostatic environment of the folding chamber. The generality as well as the precise mechanism by which substrate folding trajectories can be modulated remains to be assessed. Nonetheless, this work dictates a clear role for the termini in engaging the manifold chaperonin substrates within the crowded cellular millieu.

## Materials and Methods

The open, ATP-bound (PDB ID: 1KP8)[36] and closed ADP-bound (PDB ID: 1AON)[7] structures of GroE were simulated in implicit solvent for a total of 190 ns each. The C-terminal 23 residues were modeled into the crystal structures by hand in COOT[38]. The N-terminal methionine was not reintroduced into the structures as there was no density for it in the crystallographic electron density maps and the N-terminal methionine is often cleaved nascently by *Escherichia coli* methionine aminopeptidase. As a result, the GroEL models used here contain 547 amino acid residues per monomer. Nucleotide atoms were omitted from the simulations. However, the conformation of the atoms lining the nucleotide binding pocket were preserved as described below.

The structures were protonated and energy minimized in Gromacs[37]. Subsequently, the N-terminal 523 residues were defined as a freeze group and were fixed in space to reduce the complexity of the simulations. The two energy minimized structures were simulated in five 10 ns simulations per conformation in GBSA[39] implicit solvent and thermostatted via velocity rescaling at 370K. These simulations were subsampled to generate 19 starting conformers for each system. The starting conformations were subjected to 10 ns Langevin dynamics simulations at 370K yielding a total of 190 ns of data for each GroE conformer. The combination of high temperature with the GBSA solvation model has been shown to be predictive of protein dynamics[40,41]. All analyses presented here originate from the Langevin simulations. All simulations utilized the Amber99SB-ILDN forcefield[42]. Additional data analysis methods can be found in the supporting information. Supplemental movies were rendered in VMD[43].

## Supporting Information

S1 File Calculation of Unsymmetrized Contact Maps. Description of the calculation of the symmetrized contact maps discussed in this manuscript.
(PDF)

S1 Fig Unsymmetrized Contact Maps. The raw per-residue contact maps calculated with g_mdmat. Contact maps were calculated for each of the 38 10 ns simulations. The 19 maps corresponding to each conformation of GroE were summed to yield the maps here. Maps were normalized by dividing by the maximum value in the matrix. The open state simulation was based on PDBID:1KP8 while the closed state simulation was based on PDBID:1AON.
(TIFF)

S2 Fig Tiling of Unsymmetrized Contact Map. Tiling of the open state contact map presented in S1. Each box represents the interaction between GroEL monomer i and j within the holo complex simulation. The symmetrized contact maps in S3 were generated by summing these boxes.
(TIFF)

S3 Fig Symmetrized Contact Maps. Symmetrized maps for the open and closed state simulations of GroE. The open state was constructed from the sum of all the 547X547 blocks in the unsymmetrized map. The closed state map only reflects interactions between monomers of the cis-ring in the closed state simulation.
(TIF)

S4 Fig Comparison of Trans-apo and ATP Bound Monomers. 3D alignment of a monomer from the trans-ring of PDBID: 1AON with a monomer from the ATP-bound structure, PDBID: 1KP8. The all atom RMSD between the structures is 1.4Å. The structural alignment was generated and rendered using the PyMOL molecular graphics software.
(TIF)

S1 Movie GroEL ATP-Bound Simulation. Movie depicting twenty concatenated 10 ns simulations of a GroEL holo ring (based on PDBID: 1KP8). Residues rendered in blue were frozen throughout the course of the simulation. Orange residues are in the C-terminal region and these were allowed to move in time.
(MPG)

S2 Movie GroE Cis-Ring Simulation. Movie depicting twenty concatenated 10 ns simulations of the GroEL/ES complex (based on PDBID: 1AON). Residues rendered in blue were frozen throughout the course of the simulation. Orange residues are in the C-terminal region and these were allowed to move in time. The rendering is focused on the folding chamber referred to as the cis-ring.
(MPG)

S3 Movie GroE Trans-Ring Simulation. Movie depicting twenty concatenated 10 ns simulations of GroEL/ES in the closed state (based on PDBID: 1AON). Residues rendered in blue were frozen throughout the course of the simulation. Orange residues are in the C-terminal region and these were allowed to move in time. The rendering is focused on the trans-ring, which is in the apo configuration.
(MPG)
