# Supplementary material for: The Dynamic Conformational Cycle of the Group I Chaperonin C-Termini Revealed via Molecular Dynamics Simulation
Source: PLoS One. 2015 Mar 30;10(3):e0117724. doi: 10.1371/journal.pone.0117724 (PMC4379175; doi:10.1371/journal.pone.0117724)
Supplement: S1 File — Description of the calculation of the symmetrized contact maps discussed in this manuscript. (PDF) [file pone.0117724.s001.pdf]

Supporting Information: Calculation of Unsymmetrized Contact Maps.

Calculation of contact maps: Contact maps were generated using the program g_mdmat which is part of the Gromacs molecular dynamics software suite. The default truncation distance of 1.5 nm was utilized. Each 10 ns trajectory was submitted as a separate input for the software, generating a total of 19 contact maps per conformer. Contact scores were calculated on a per-residue basis. Contact maps for each of the open and closed conformers were subsequently combined by matrix addition. The maps were then normalized such that the largest value in any array was unity. The resulting maps are depicted in Fig S1.

Symmetrization of contact maps to yield contact scores: Contact maps for the full GroE complexes (Fig S1) were symmetrized in order to account for interactions between different monomers of the same complex. To carry out the symmetrization, each 547x547 submatrix (C_{ij} in Fig S2) was summed and normalized such that the largest value the matrix became unity (Fig S3).

$$M = \frac{1}{max\left(\sum_{i=1}^{14}\sum_{j=1}^{14} C_{ij}\right)} \sum_{i=1}^{14}\sum_{j=1}^{14} C_{ij}$$

In the case of the closed conformer, the submatrices corresponding to the seven GroEL subunits in the GroES bound ring were summed separately from the seven subunits corresponding to the trans-ring.
