# Supplementary figures and images for: The Dynamic Conformational Cycle of the Group I Chaperonin C-Termini Revealed via Molecular Dynamics Simulation
Source: PLoS One. 2015 Mar 30;10(3):e0117724. doi: 10.1371/journal.pone.0117724 (PMC4379175; doi:10.1371/journal.pone.0117724)

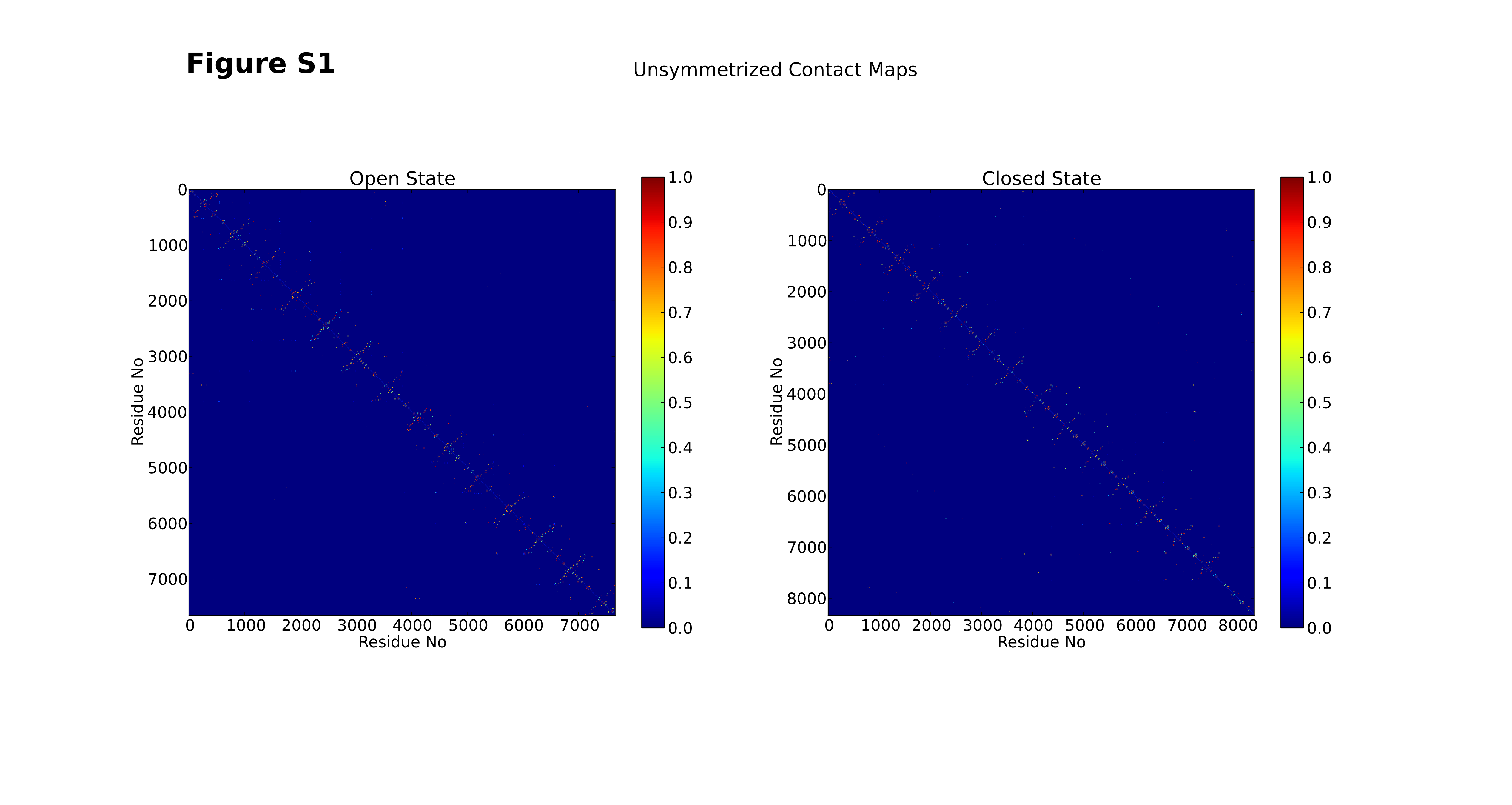

Supplement: S1 Fig — The raw per-residue contact maps calculated with g_mdmat. Contact maps were calculated for each of the 38 10 ns simulations. The 19 maps corresponding to each conformation of GroE were summed to yield the maps here. Maps were normalized by dividing by the maximum value in the matrix. The open state simulation was based on PDBID:1KP8 while the closed state simulation was based on PDBID:1AON. (TIFF) [file pone.0117724.s002.tiff]

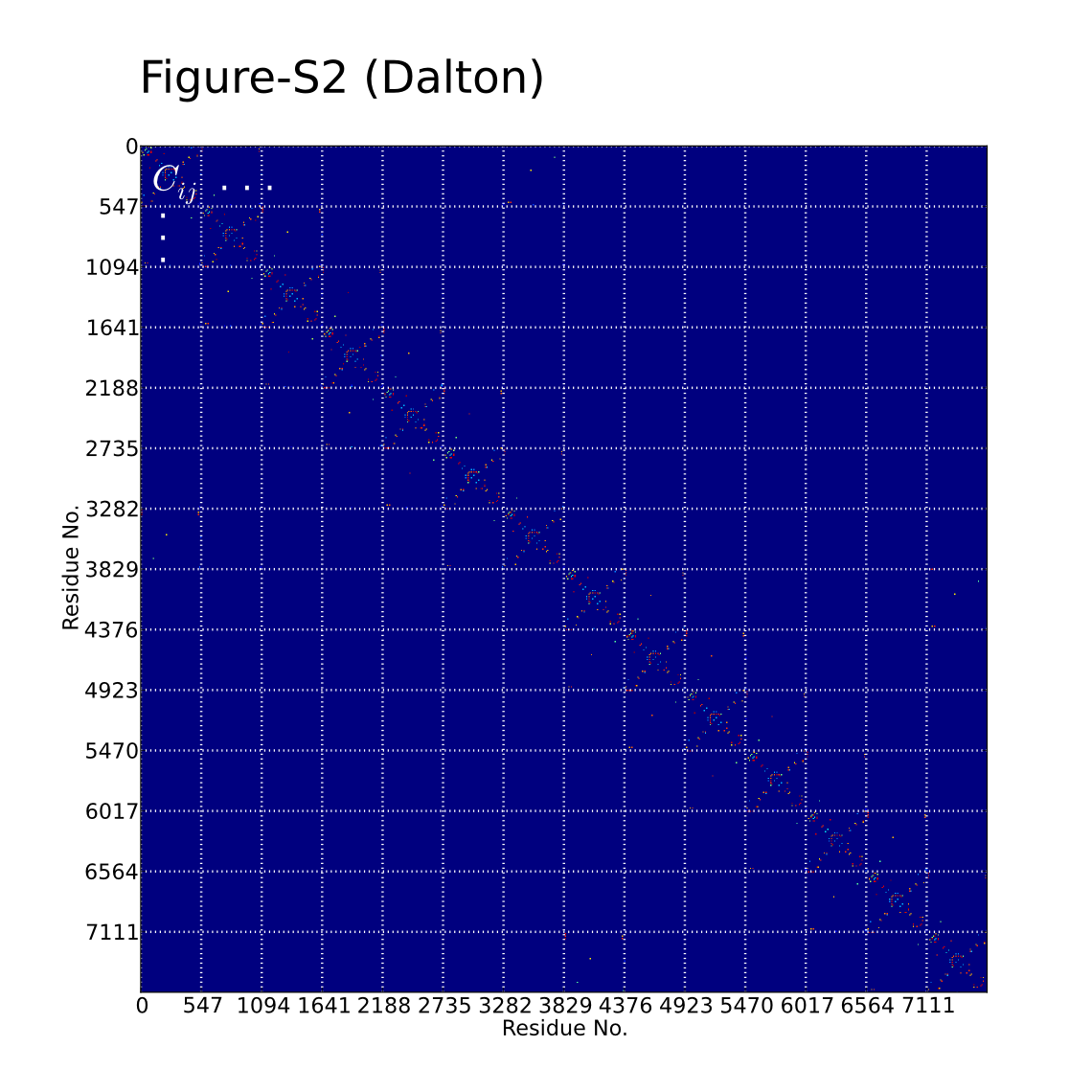

Supplement: S2 Fig — Tiling of the open state contact map presented in S1. Each box represents the interaction between GroEL monomer i and j within the holo complex simulation. The symmetrized contact maps in S3 were generated by summing these boxes. (TIFF) [file pone.0117724.s003.tiff]

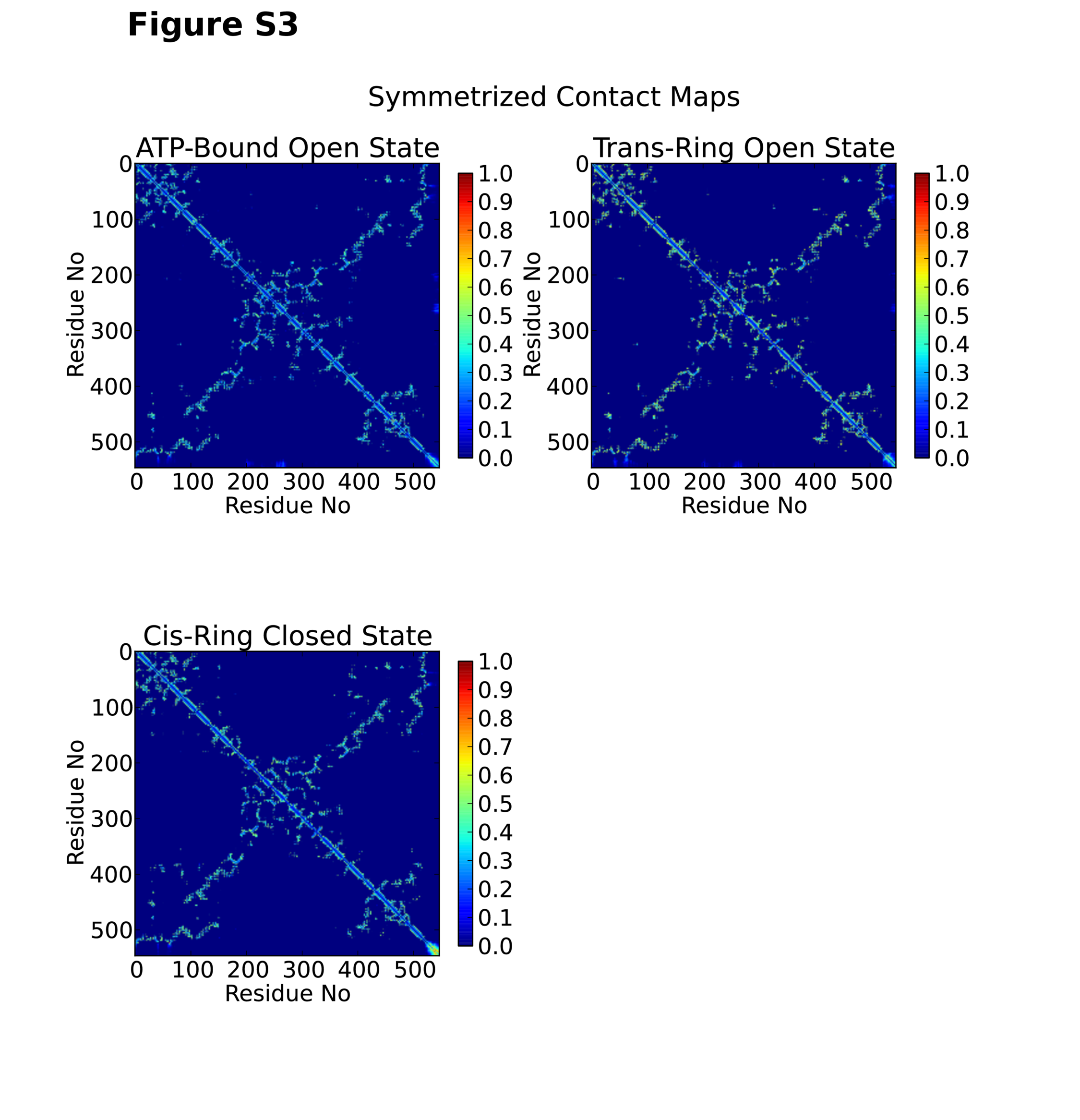

Supplement: S3 Fig — Symmetrized maps for the open and closed state simulations of GroE. The open state was constructed from the sum of all the 547X547 blocks in the unsymmetrized map. The closed state map only reflects interactions between monomers of the cis-ring in the closed state simulation. (TIF) [file pone.0117724.s004.tif]

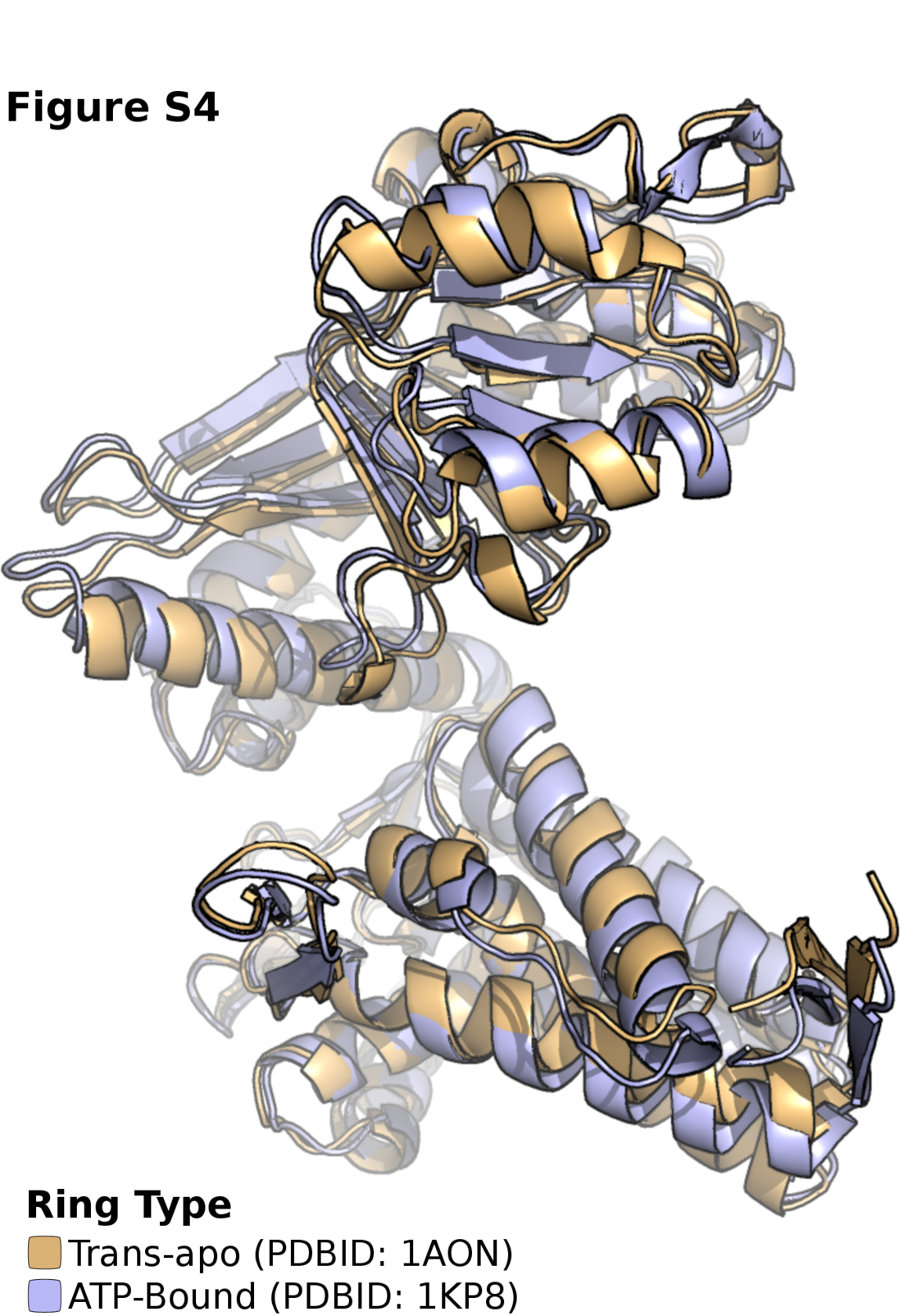

Supplement: S4 Fig — 3D alignment of a monomer from the trans-ring of PDBID: 1AON with a monomer from the ATP-bound structure, PDBID: 1KP8. The all atom RMSD between the structures is 1.4Å. The structural alignment was generated and rendered using the PyMOL molecular graphics software. (TIF) [file pone.0117724.s005.tif]
